# On Closure of the Pupil in Iritis

**Published:** 1825-08

**Authors:** Wm. Mackenzie

**Affiliations:** Andersonian Professor of Anatomy and Surgery, and one of the Surgeons to the Glasgow Eye Infirmary.


**Art. VI.—*On Closure of the Pupil in Iritis.*** By **Wm. Mackenzie,**

Andersonian Professor of Anatomy and Surgery, and one of the Surgeons to the Glasgow Eye Infirmary.

Although obliteration of the pupil is by far the most important symptom in iritis, hitherto no explanation has been offered of this remarkable morbid change.

The mere inflamed state of the iris seems quite insufficient to explain it. The effusion of fibrin into the posterior chamber is sufficient to account for the pupil not opening again, after it is once closed, but does not account for the closure in the first instance. Intolerance of light is not the cause, for in general this is not an attendant on iritis. Inflammation of the retina is not the cause; for, although occasionally the inflammation in iritis does spread to the more deeply-seated textures of the eye, it is much more common to see the pupil completely obliterated, while the retina continues sound, so that vision may afterwards be restored by the formation of an artificial pupil.

In the Lectures on the Structure and Diseases of the Eye, which I have given for the last seven years, I have ventured to ascribe the closure of the pupil in iritis to the fact, that the natural state of that opening (the state into which it falls during the insensibility of sleep,) is closure, not expansion. The discovery of this fact we owe to Fontana;* its truth has been acknowledged by Janin† and Cuvier,‡ and may easily be ascertained by any inquirer. Approach a child fast asleep, and suddenly raise the upper eyelid; the pupil will appear all but closed, and only gradually, as the state of sleep is broken, will it be seen first of all to expand, and at last to accommodate itself to the degree of light to which the eye is exposed.

* *Dei Moti dell' Iride.* Lucca, 1765; page 21.

† *Mémoires sur l'Œil.* Lyon, 1772; page 8.

‡ *Leçons d'Anatomie Comparée,* tome ii. page 409.

Iritis is always aggravated, and often appears to commence, during the night, particularly after the operations for cataract. The probable cause of this is the contraction of the pupil, or rather the expansion of the iris, during sleep. Hence the propriety of applying the extract of belladonna to the forehead every evening, particularly in cases of incipient iritis.

If no means are used to counteract the progress of iritis, the pupil is observed to become, day after day, smaller and smaller, and less and less dilatable, till it be completely closed and bound down by effused fibrin to the anterior hemisphere of the crystalline capsule. If belladonna only be employed, without the detraction of blood and the exhibition of mercury, the pupil will be kept dilated; and, if the narcotic has been applied early in the disease, the pupil will be widely expanded, and in this state will become adherent to the crystalline capsule. The detraction of blood, to lower the sanguiferous action,—the use of mercury, to prevent the effusion of fibrin, or to bring about its absorption, if already effused,—and the application of belladonna, to counteract the return of the iris to its natural state of rest, relaxation, and expansion, during sleep, makes up, then, the chief parts of the treatment of iritis, whether the disease be traumatic, atmospheric, or syphilitic; and the belladonna ought to be applied, not irregularly or merely every second or third day, but regularly every evening.

*Spreull's-court, Glasgow; June 1st,* 1825.

